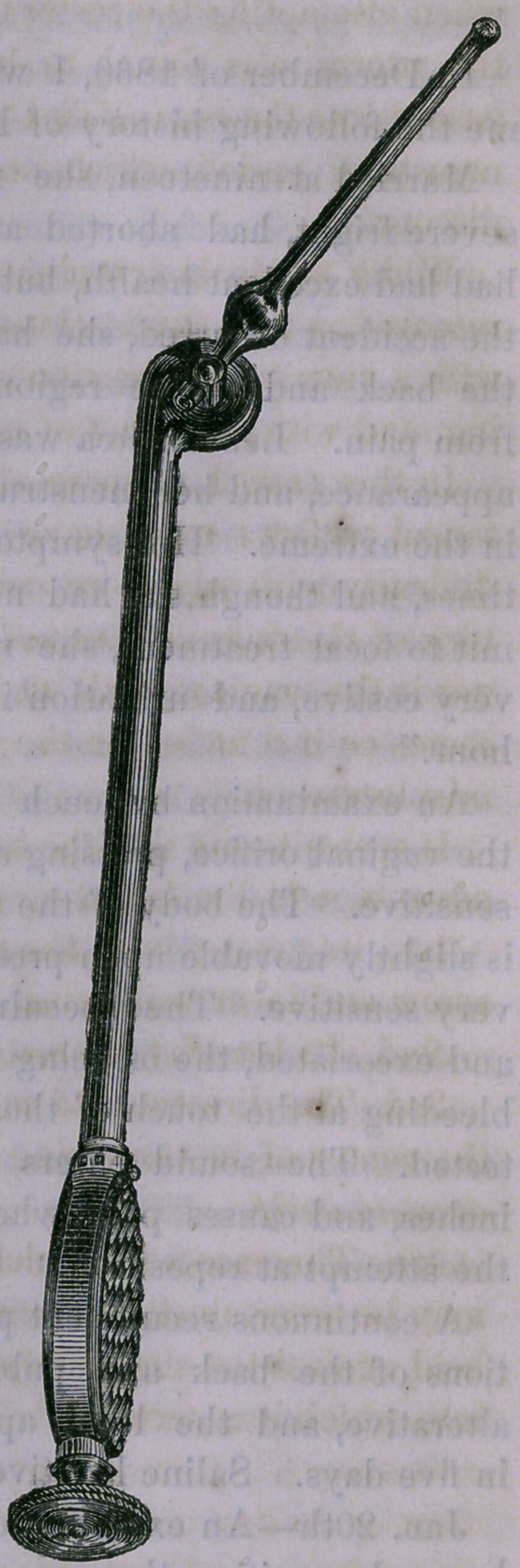# Retroversion of the Uterus, with a Description of an Instrument Designed for Its Reposition

**Published:** 1861-04

**Authors:** H. W. Jones


### RETROVERSION OF THE UTERUS,

### WITH A DESCRIPTION OF AN INSTRUMENT DESIGNED FOR ITS REPOSITION.

BY H. W. JONES, M. D.

In December of 1860, I was called to see a lady who gave me the following history of her case:

Married at nineteen, she was soon pregnant, and, after a severe fright, had aborted at the third month Previously had had excellent health, but since the summer of 1859, when the accident occurred, she had experienced much suffering in the back and pelvic regions, being at no time entirely free from pain. Leucorrhœa was abundant, of a "white of egg" appearance, and her menstrual returns were dysmenorrhœic in the extreme. Her symptoms were all aggravated at such times, and though she had never been willing, before, to submit to local treatment, she now desires it. The bowels are very costive, and urination is required "at least every half hour."

An examination by touch detects the os uteri just within the vaginal orifice, pressing against the urethra, patulous and sensitive. The body of the uterus fills the sacral cavity and is slightly movable upon pressure. The parts are everywhere very sensitive. The speculum discloses the cervix tumefied and excoriated, the os being filled with tenacious mucus, and bleeding at the touch of the sponge. No tumors can be detected. The sound enters the uterine cavity nearly three inches, and causes pain, when in contact with the fundus, in the attempt at reposition.

A continuous recumbent position is advised, with cool ablutions of the back and pubic regions, a continued mercurial alterative, and the local application of nitrate of silver once in five days. Saline laxatives are to be used *pro re nata.*

Jan. 20th—An examination now finds the cervix less swollen and sensitive, the excavations healed, and the os smaller, discharging but little mucus. The body of the uterus and the walls of its cavity are much less sensitive. I attempted, at this visit, to replace the organ, while the patient was upon her knees and elbows. Both finger and sound were used perseveringly, but with no effect upon the uterus.

I then devised the instrument, (here shown at half size) which was made from my drawings by Messrs. Degenhardt & Loewe, of this city. It consists of a handle, shaft and stem, the latter moving in the arc of a circle in either direction and governed by a screw at the end of the handle.

The mechanical principle involved is the same with that used at present in the apparatus for turning the guitar.

The stem may be placed at any desired angle with the shaft and introduced as in the use of the sound. Then, by means of the screw, the uterus may be gently and completely replaced in all ordinary cases.

In the case related, this *Uterine Retractor* was used four times at intervals of forty-eight hours, firm, but careful, pressure being exerted and persevered in for several minutes. Previous to its withdrawal, I introduced a colpornyter into the *rectum*, which was left in situ for three or four hours, and then removed by the patient herself.

The effect of these measures was hardly perceptible at first, but on the third occasion of their use, the uterus rose to the sacral promontory, and on the fourth a complete reposition was effected. More or less pain always attended the operation, but the patient declared that it was less than I had caused when attempting the restoration, by Simpson's sound. After the uterus was found to be in place, the colporynter was passed into the *vagina* and was worn constantly until the next menstrual period, which occurred with comparatively slight discomfort.

While the local symptoms have thus been almost entirely removed, a few applications of the silver have been made, with a view to a correction of the slight hypertrophy remaining, and with the effect of curing the leucorrhœa.

In this case, I suppose slight adhesions of opposed peritoneal surfaces must have existed in order to account for the difficulty with which reposition was secured. The promontory of the sacrum does not encroach upon the superior strait, nor is the vaginal wall at all shortened. At present there seems no inclination on the part of the uterus to return to its misplacement.

It seems to me that the instrument described possesses great advantage in the following points:

1st. Its great power, the resultant of the combination of the screw and lever principles.

2nd. Its security against injury of the uterine cavity.

3rd. The directness of motion communicated to the uterus, the center of motion being *within the vagina*, and *at the very os uteri itself.*

4th. The ease with which the exact position of the uterus may be ascertained at any stage of its progress, by the finger held against the slight projection of the stem through the axle into which it is screwed.

## Figures and Tables

**Figure f1:**